# Evidence for a Robertsonian fusion in *Solea senegalensis* (Kaup, 1858) revealed by zoo-FISH and comparative genome analysis

**DOI:** 10.1186/s12864-018-5216-6

**Published:** 2018-11-14

**Authors:** Aglaya García-Angulo, Manuel A. Merlo, Silvia Portela-Bens, María E. Rodríguez, Emilio García, Ahmed Al-Rikabi, Thomas Liehr, Laureana Rebordinos

**Affiliations:** 1 Área de Genética, Facultad de Ciencias del Mar y Ambientales, Universidad de Cádiz, 11510 Cádiz, Spain; 2 Institut für Humangenetik, Universitätsklinikum Jena, 07743 Jena, Germany

**Keywords:** Comparative chromosome painting, Chromosome fusion, Chromosome evolution, Pleuronectiformes, Senegalese sole

## Abstract

**Background:**

*Solea senegalensis* (Kaup, 1858) is a commercially important flatfish species, belonging to the Pleuronectiformes order. The taxonomy of this group has long been controversial, and the karyotype of the order presents a high degree of variability in diploid number, derived from chromosomal rearrangements such as Robertsonian fusions. Previously it has been proposed that the large metacentric chromosome of *S. senegalensis* arises from this kind of chromosome rearrangement and that this is a proto-sex chromosome.

**Results:**

In this work, the Robertsonian origin of the large metacentric chromosome of *S. senegalensis* has been tested by the Zoo-FISH technique applied to two species of the Soleidae family (*Dicologlossa cuneata* and *Dagetichthys lusitanica)*, and by comparative genome analysis with *Cynoglossus semilaevis*. From the karyotypic analysis we were able to determine a chromosome complement comprising 2n = 50 (FN = 54) in *D. cuneata* and 2n = 42 (FN = 50) in *D. lusitanica.* The large metacentric painting probe gave consistent signals in four acrocentric chromosomes of the two Soleidae species; and the genome analysis proved a common origin with four chromosome pairs of *C. semilaevis*. As a result of the genomic analysis, up to 61 genes were annotated within the thirteen Bacterial Artificial Chromosome clones analysed.

**Conclusions:**

These results confirm that the large metacentric chromosome of *S. senegalensis* originated from a Robertsonian fusion and provide new data about the chromosome evolution of *S. senegalensis* in particular, and of Pleuronectiformes in general.

**Electronic supplementary material:**

The online version of this article (10.1186/s12864-018-5216-6) contains supplementary material, which is available to authorized users.

## Background

The Pleuronectiformes order comprises more than 700 species belonging to 123 genera and 11 families, distributed worldwide [1].The taxonomic status of the Pleuronectiformes order has been discussed at length by several authors, some supporting a monophyletic [2–4], others a poly/paraphyletic origin of the group [5–7]. This controversy centres on two suborders, i.e. Pleuronectoidei and Psettoidei, and is based on the rapid adaptive radiation and major genomic reorganizations that suggest different strategies in the adaptation to benthic life [8].

The group comprises commercially-important species, highly appreciated by consumers and hence a profitable group for exploitation. The main flatfish species produced by aquaculture are turbot (*Scophthalmus maximus*), Japanese flounder (*Paralichthys olivaceus*), tongue sole (*Cynoglossus semilaevis*), Atlantic halibut (*Hippoglossus hippoglossus*), Senegalese sole (*Solea senegalensis*) and common sole (*S. solea*). A characteristic attribute of this order is the flat morphology of these fishes and the exceptional variability observed in the karyotype, with chromosomal numbers ranging from 2n = 26, observed in the Paralichthyidae *Citarichthys spilopterus*, to 2n = 48, found in most of Pleuronectidae species [9]. This variability has been explained by the occurrence of Robertsonian fusions throughout the evolution of Pleuronectiformes [8].

*S. senegalensis* possess 2n = 42 chromosomes, where three pairs are metacentric, two are sub-metacentric, four pairs are sub-telocentric and twelve are acrocentric [10] and it has been thought that the major metacentric pair originated from a Robertsonian fusion [11].

Both the commercial interest in Pleuronectiformes, and the taxonomic controversy over the species have contributed to a considerable increase in the studies about these species from physiological, molecular, cytogenetic and genomic perspectives. Genomic approaches characterise most of the research published in recent years (reviewed by [8]). However, cytogenetic information has also been widely used to resolve the Pleuronectiformes taxonomy [9, 12, 13] and as support for genomic results [14, 15].

On *S. senegalensis*, a wide variety of work reporting both cytogenetic and genome information has been published in the last decade. The genome mapping of this species started with the localization of the minor and major ribosomal genes, both co-localized in a submetacentric pair and another additional 5S rDNA signal in an acrocentric pair [16, 17]. Moreover, repetitive sequences, as (GATA)_n_ and telomeric (TTAGGG)_n_ were hybridized, resulting in dispersed and telomeric localization, respectively [17]. The elaboration of a Bacterial Artificial Chromosome (BAC) library on *S. senegalensis* has allowed single copy genes to be localized [18] and enabled the cytogenetic map to be integrated with the physical map obtained by BAC-sequencing [11, 19, 20]. Other achievements reported in *S. senegalensis* include the complete sequence of the mitochondrial genome [21], construction of a BAC library [19], a genetic linkage map [22] and the transcriptome [23].

The Pleuronectiformes order encompasses a wide range of karyotype sizes, ranging from 2n = 26 to 2n = 48 chromosomes (reviewed by [9]). Considering this high karyotype variability, the complex taxonomy of the Pleuronectiformes order and the latest available data, it can be stated that chromosome fusion has probably directed the evolution of this group. To confirm definitively this assumption, for the first time, a cross-species chromosome painting (Zoo-FISH) technique has been applied to several different species of the Soleidae family (*S. senegalensis*, *Dicologlossa cuneata* and *Dagetichthys lusitanica*), using as probe the largest metacentric pair from female individuals of *S. senegalensis*, because this species has been proposed to have a XX/XY sex determination system and this chromosome has also been proposed as a proto-sex chromosome. Furthermore, an exhaustive comparison has been made of the genes presented in each arm of the metacentric pair, between *S. senegalensis* and a flatfish species that belongs to a different family, specifically the Cynoglossidae (*C. semilaevis*).

## Material and methods

### Obtention of chromosome preparations and karyotypes

The *S. senegalensis* biological samples were obtained from the Central Research Services in Marine Culture (SCI-CM) of the University of Cádiz, while individuals of *D. cuneata* and *D. lusitanica* were captured wild. The chromosome preparations of *S. senegalensis* were obtained from colchicine-treated larvae according to [20]. Chromosome preparations of *D. cuneata* and *D. lusitanica* were obtained from spleen and anterior kidney culture, in which fishes were first anesthetized with clove oil (40 mg/L), after that the individuals were injected intraperitoneally with colchicine 0.05% and kept in an oxygenated tank for 3–4 h. Afterwards, the fishes were sacrificed with a clove oil overdose and spleen and anterior kidney were extracted and broken up in a 0.056% KCl solution. This cellular solution was filtered in a cell strainer from 100 to 40 μm. Finally, cells were subjected to hypotonic shock with a KCl solution and fixed in Carnoy solution. The experimental procedures are according to the recommendation of the University of Cádiz (Spain) for the use of laboratory animals and the Guidelines of the European Union Council (86/609/EU).

Karyotyping was performed using conventional staining techniques with Giemsa (10% in phosphate buffer pH 6,8). The chromosomes were measured using the GIMP 2.8.22 program and, after that, were paired and grouped according to the classification described by [24] based on the relative length (RL), centromeric index (CI) and arm ratio (AR).

### Isolation, sequencing and annotation of BAC clones

BAC clones were isolated using the Large Construct Kit (Qiagen, Hilden, Germany), then were sent to be sequenced by the Illumina sequencing platform (Illumina, San Diego, California, USA) (Accession Numbers AC278047-AC278120). The functional and structural annotations of the gene sequences identified in each BAC were carried out in a semi-automated process. Proteins and Expressed Sequence Tag (EST) from *S. senegalensis* and related species were compared. The homologous sequences obtained were used to get the best predictions for gene annotation. Finally, all available information was used to create plausible models and, when possible, functional information was added. Using the Apollo genome editor [25], Signal map software (Roche Applied Science, Penzberg, Germany), and Geneious R11 [26], the results were individually completed and adjusted in the final edition process of the annotation. In addition, a search for repetitive elements was carried out with the RepeatMasker program [27].

### Comparative genomic analysis

All the genes annotated in the putative chromosome derived from a Robertsonian fusion were used for comparative genomic analysis. For this purpose, genomic information was extracted from the National Center for Biotechnology Information (NCBI) database to compare with *C. semilaevis*, as the flatfish reference genome. In addition, the data was used to identify reorganizations within the chromosomes.

### Chromosome microdissection

The chromosome suspensions were dropped onto pre-cleaned coverslips and incubated in Giemsa solution. The microdissection was performed using an inverted microscope (Zeiss Axiovert 135) with a mechanical micromanipulator. Sixteen copies of the largest metacentric were microdissected from the female karyotype of *S. senegalensis* using sterile microneedles and micropipettes with 20 μl of collection drop solution (30% glycerol, 10 mM Tris/HCl, pH 7.5, 10 mM NaCl, 0.1% SDS, 1 mM EDTA, 0.1% Triton X-100, 1.44 mg/ml proteinase K). Micropipettes were put into a humidified tray at 60 °C and, afterwards, the solutions were transferred to 0.5 ml tubes.

### Multiple FISH and chromosome painting

To prepare Fluorescence in situ Hybridation (FISH) probes, BAC clones were grown on Luria Bertani (LB) broth containing chloramphenicol at 37 °C, overnight. BAC-DNA was extracted using the BACMAX™ DNA purification kit (Epicentre Biotechnologies, Madison, USA), following the manufacturer’s instructions. The presence of the insert was evaluated by digestion with *Eco* RI and agarose gel electrophoresis (0.8%).

The BAC clones and large metacentric chromosome were amplified by Degenerate Oligonucleotide Primed - Polymerase Chain Reaction (DOP-PCR) and then labelled by a conventional PCR using four different fluorochromes, i.e. Texas Red (TR) (Life Technologies, Carlsbad, California, USA), Spectrum Orange (SO), Fluorescein isothiocyanate (FITC) (Abbott Molecular/ENZO, Illinois, USA), and diethylaminocoumarin (DEAC) (Vysis, Downers Grove, USA), using the protocol described by [28].

Chromosome preparations were pre-treated with pepsin solution at 37 °C and fixed with paraformaldehyde solution. Finally, the preparations were dehydrated with ethanol series of 70, 90, and 100%, and air-dried. Hybridization and post-hybridization treatment were according to [20]. FISH with painting probes was performed on female and male chromosome preparations of *S. senegalensis,* female of *D. lusitanica* and male of *D. cuneata*.

The slides were visualized with a fluorescence microscope (Olympus BX51 and/or Zeiss Axioplan using software of MetaSystems, Altlussheim, Germany) equipped with a digital CCD camera (Olympus DP70) to take the pictures.

## Results

**Fig. 1:**
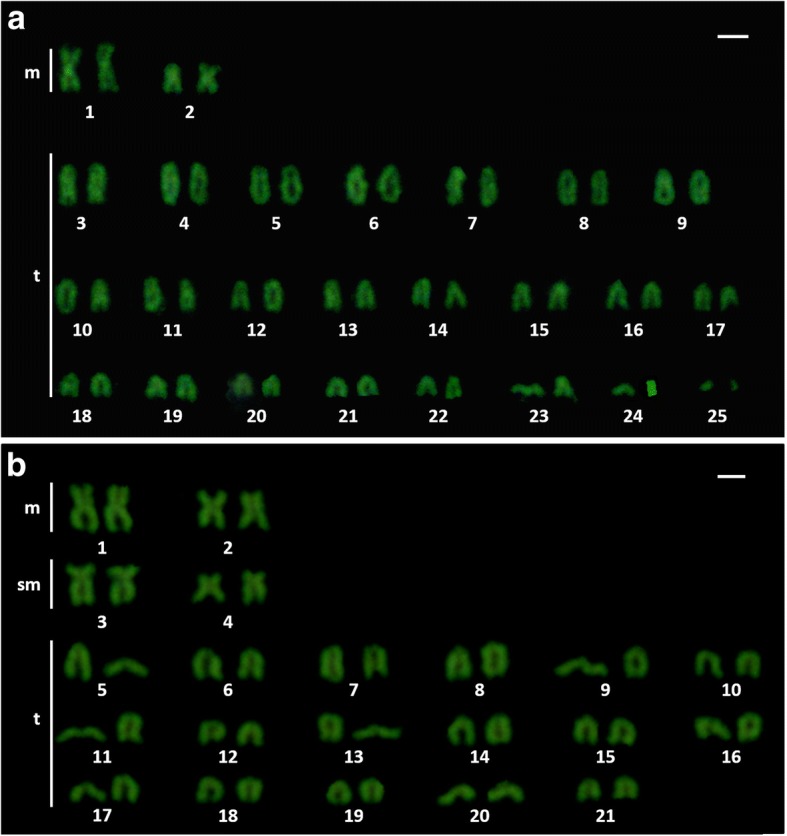
**a** Karyotype of *Dicologlossa cuneata* (2n = 50). **b** Karyotype of *Dagetichthys lusitanica* (2n = 42). Scale bar = 1 μm

As described by [10], the karyotype of *S. senegalensis* is 2n = 42 (Fundamental Number FN = 60), with 6 M + 4SM + 8ST + 24 T. Meanwhile, *C. semilaevis* has a karyotype 2n = 42 acrocentric chromosomes (FN = 42) [29]. The result of the karyotype analysis allows the determination of a chromosome complement comprising 2n = 50 (FN = 54) in *D. cuneata* and 2n = 42 (FN = 50) in *D. lusitanica* (Fig. 1). The karyotype formula is 4 m + 46 t and 4 m + 4sm + 34 t, for *D. cuneata* and *D. lusitanica* respectively.

**Fig. 2:**
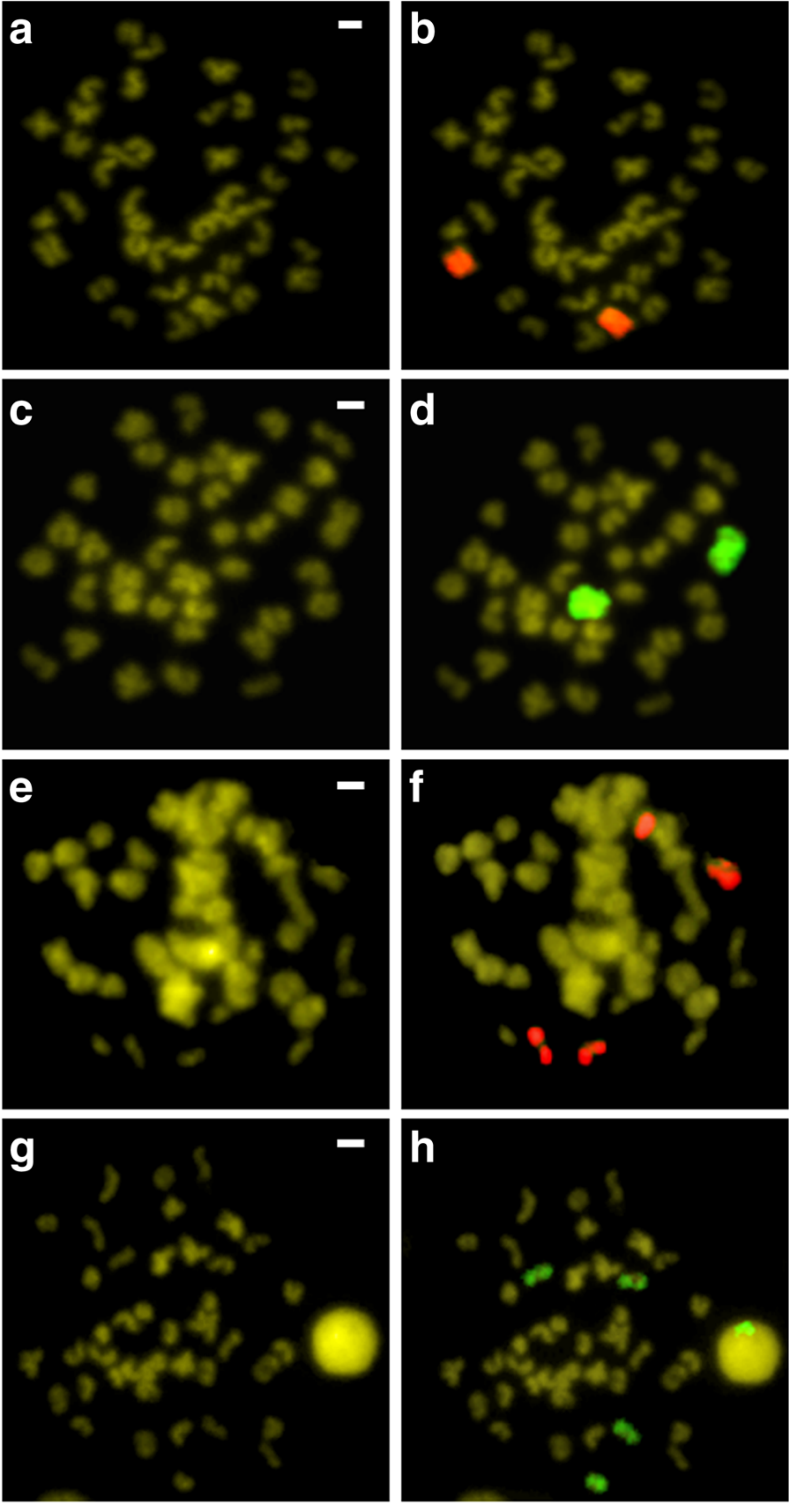
Chromosome painting using as probe the large metacentric chromosome pair of *Solea senegalensis*: **a, b** female of *S. senegalensis*; **c, d** male of *S. senegalensis.* Zoo- FISH of the *S. senegalensis* large metacentric chromosome in: **e, f** *Dicologlossa cuneate*; **g, h** *Dagetichthys lusitanica*. Scale bar = 1 μm

The chromosome painting probe highlighted in full the large metacentric pair of *S. senegalensis*, both female (Fig. 2a, b) and male (Fig. 2c, d). This same probe painted two acrocentric chromosomes in both *D. cuneata* (Fig. 2e, f) and *D. lusitanica* (Fig. 2g, h). However, in these two species, centromeric regions did not appear painted (Fig. 2f, h).

**Fig. 3:**
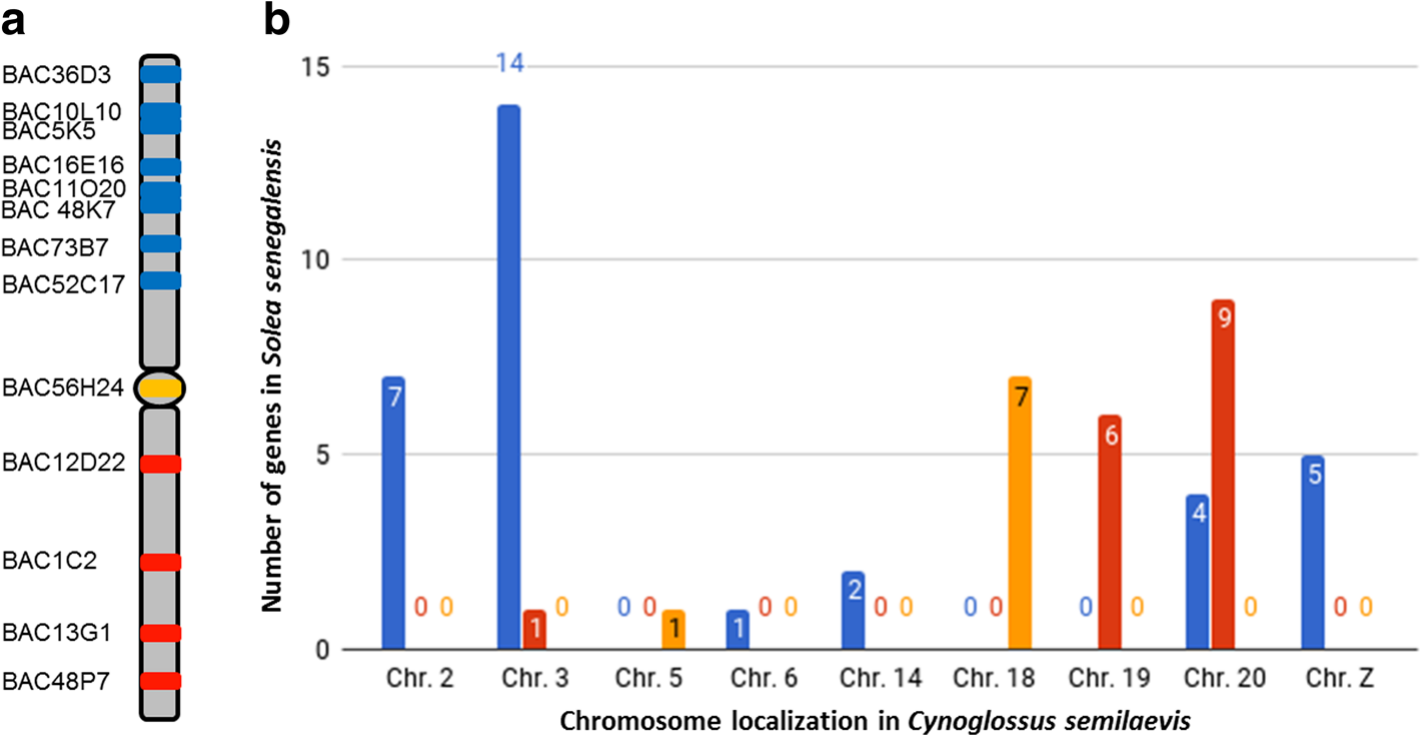
**a** BAC clones localization within each arm of the *Solea senegalensis* large metacentric chromosome. **b** Localization of the genes contained within BAC clones among the *Cynoglossus semilaevis* chromosomes. Blue bars denote the number of genes located in arm 1 of *S. senegalensis* that were found in the *C. semilaevis* chromosome shown in the X-axis. Red bars denote the number of genes located in arm 2 of *S. senegalensis* that were found in the *C. semilaevis* chromosome shown in the X-axis. Yellow bars denote the number of genes located in the centromeric position of *S. senegalensis* that were found in the *C. semilaevis* chromosome shown in the X-axis

**Fig. 4:**
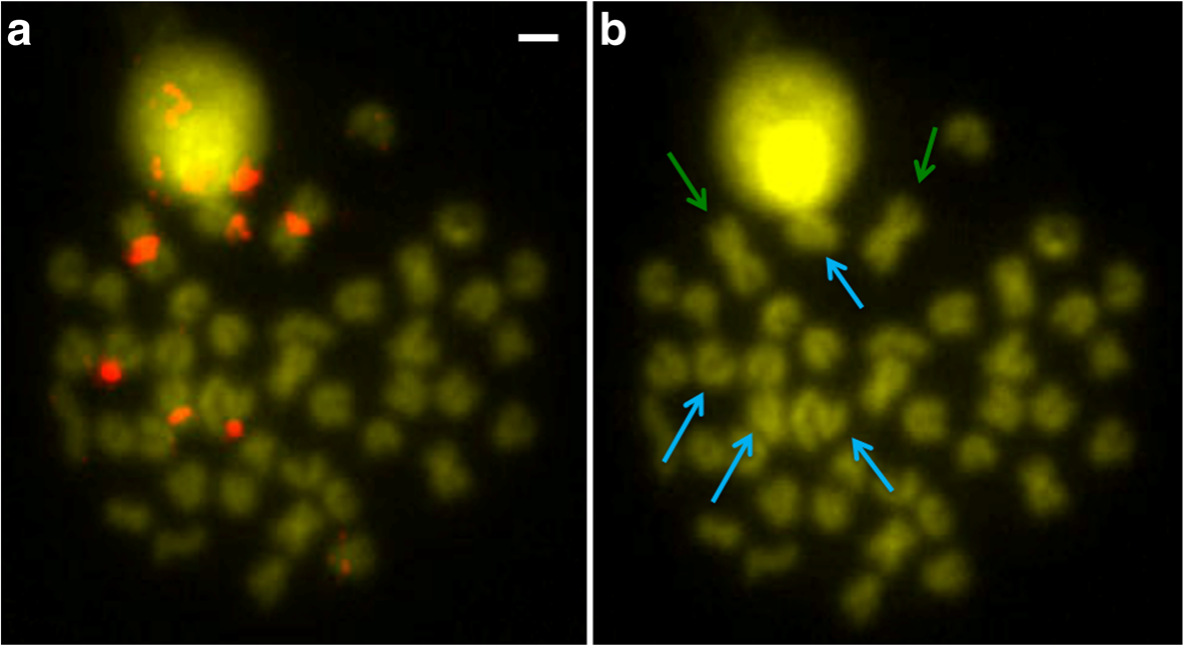
**a** BAC56H24 chromosome localization. **b** Same plate without signals to show better the chromosome morphology. Green arrows indicate the large metacentric chromosome. Cyan arrows indicate subtelocentric and acrocentric chromosomes that also presented hybridization signals. Scale bar = 1 μm

A total of 13 BAC clones were localized in the largest metacentric pair of *S. senegalensis* and 61 different genes were annotated within them (Table 1). The multiple BAC-FISH allowed the localization of all these BAC clones in one arm or the other, although the similarity in size of the two chromosome arms made it difficult to differentiate between the q and p arms. Hence, the BAC clones were distributed between arm 1 and arm 2 (Fig. 3a, Additional files 1 and 2). Thus, BAC5K5, BAC10L10, BAC11O20, BAC16E16, BAC36D3, BAC48K7 and BAC52C17 were localized in arm 1; whereas, BAC1C2, BAC12D22, BAC13G1 and BAC48P7 were localized in arm 2. However, BAC56H24 could not be localized in a specific arm, because the hybridization signal was at the centromere, not only of the large metacentric pair, but also in two other chromosome pairs, subtelocentric and acrocentric respectively (Fig. 4).

**Table 1:** BAC clones found in the large metacentric chromosome and gene annotation

BAC	Gene annotation
BAC1C2	*neurobeachin* (***nbea***)
BAC5K5	*Tropomyosin alpha-4 chain* (***tpm4***); *Krueppel-like factor 2* (***klf2***); *Epidermal growth factor receptor substrate like 1* (***eps15l1***); *Ras-related protein Rab-8A* (***rab8a***); *Calcium and integrin-binding family member 3* (***cib3***); *Excitatory amino acid transporter 1* (***slc1a3***); *Histone H2A* (***H2a***); *Histone H3* (***H3***); *Histone H4* (***H4***); *Histone H2B* (***H2b***); *Histone H1* (***H1***); *Calreticulin* (***calr***); *Retinal homeobox protein Rx2* (***rx2***); *Tropomyosin alpha-1 chain* (***tpm1***); *AP-1 complex subunit mu-1* (***ap1m1***)
BAC10L10	*Krueppel-like factor 2* (***klf2***); *Epidermal growth factor receptor substrate 15-like 1* (***eps15l1***); *Retinal homeobox protein Rx2* (***rx2***); *Calreticulin* (***calr***)
BAC11O20	*Arrestin domain-containing protein 3* (***arrdc3***); *Aquaporin-3* (***aqp3***); *Nucleolar protein 6* (***nol6***)
BAC12D22	*Histone H2A* (***H2a***); *Histone H3* (***H3***); *Histone H4* (***H4***); *Histone H2B* (***H2b***); *Histone H1* (***H1***); *Ankyrin repeat domain-containing protein 45* (***ankrd45***); *Transmembrane protein 70, mitochondrial* (***tmem70***)
BAC13G1	*WW domain-containing adapter protein with coiled-coil* (***wac***)
BAC16E16	*Doublesex and mab-3-related transcription factor 2* (***dmrt2***); *Doublesex and mab-3-related transcription factor 3* (***dmrt3***)
BAC36D3	*melanocortin receptor 4* (***mc4r***)
BAC48K7	*Doublesex and mab-3-related transcription factor 3* (***dmrt3***); *KN motif and ankyrin repeat domain-containing protein 1* (***kank1***); *Doublesex and mab-3-related transcription factor 1* (***dmrt1***); *fructose-1,6-bisphosphatase 1* (***fbp1***); *cilia- and flagella-associated protein 157* (***cfap157***); *Doublesex and mab-3-related transcription factor 2* (***dmrt2***)
BAC48P7	*7-alpha-hydroxycholest-4-en-3-one 12-alpha-hydroxylase* (***cyp8b1***); *parathyroid hormone/parathyroid hormone-related peptide receptor* (***pth1r***); *myosin light chain 3* (***myl3***); *corticotropin-releasing factor receptor 2* (***crhr2***); *rho-associated protein kinase 1* (***rock1***); *ubiquitin carboxyl-terminal hydrolase 14* (***usp14***); *aquaporin-1* (***aqp1***); *THO complex subunit 1* (***thoc1***)
BAC52C17	*acyl-protein thioesterase 1* (***lypla1***); *N-acetyltransferase ESCO1* (***esco1***); *vacuolar protein sorting-associated protein 41 homolog* (***vps41***); *transcription factor Sox-17-alpha* (***sox17a***); *oxygen-regulated protein 1* (***rp1***); *39S ribosomal protein L15, mitochondrial* (***mrpl15***); *Regulator of G-protein signaling 20* (***rgs20***); *Myosin regulatory light chain 2, smooth muscle* (***mlc2***); *FAST kinase domain-containing protein 3, mitochondrial* (***fastkd3***); *charged multivesicular body protein 5* (***chmp5***); *myomesin-1* (***myom1***); *kappa-type opioid receptor* (***oprk1***)
BAC56H24	*A-kinase anchor protein 9* (***akap9***); *potassium/sodium hyperpolarization-activated cyclic nucleotide-gated channel 4* (***hcn4***); *aquaporin-10* (***aqp10***); *HCLS1-associated protein X-1* (***hax1***); *tuftelin* (***tuft1***); *ubiquitin-associated protein 2-like* (***ubap2l***); *zinc finger protein 687b* (***znf687b***); *uncharacterized protein C1orf43 homolog* (***C1orf43***); *phosphatidylinositol 4-phosphate 5-kinase type-1 alpha* (***pip5k1a***)
BAC73B7	*rho GTPase-activating protein 21 (* ***arhgap21*** *); apolipoprotein D (* ***apod*** *); otospiralin (* ***otos*** *)*

**Fig. 5:**
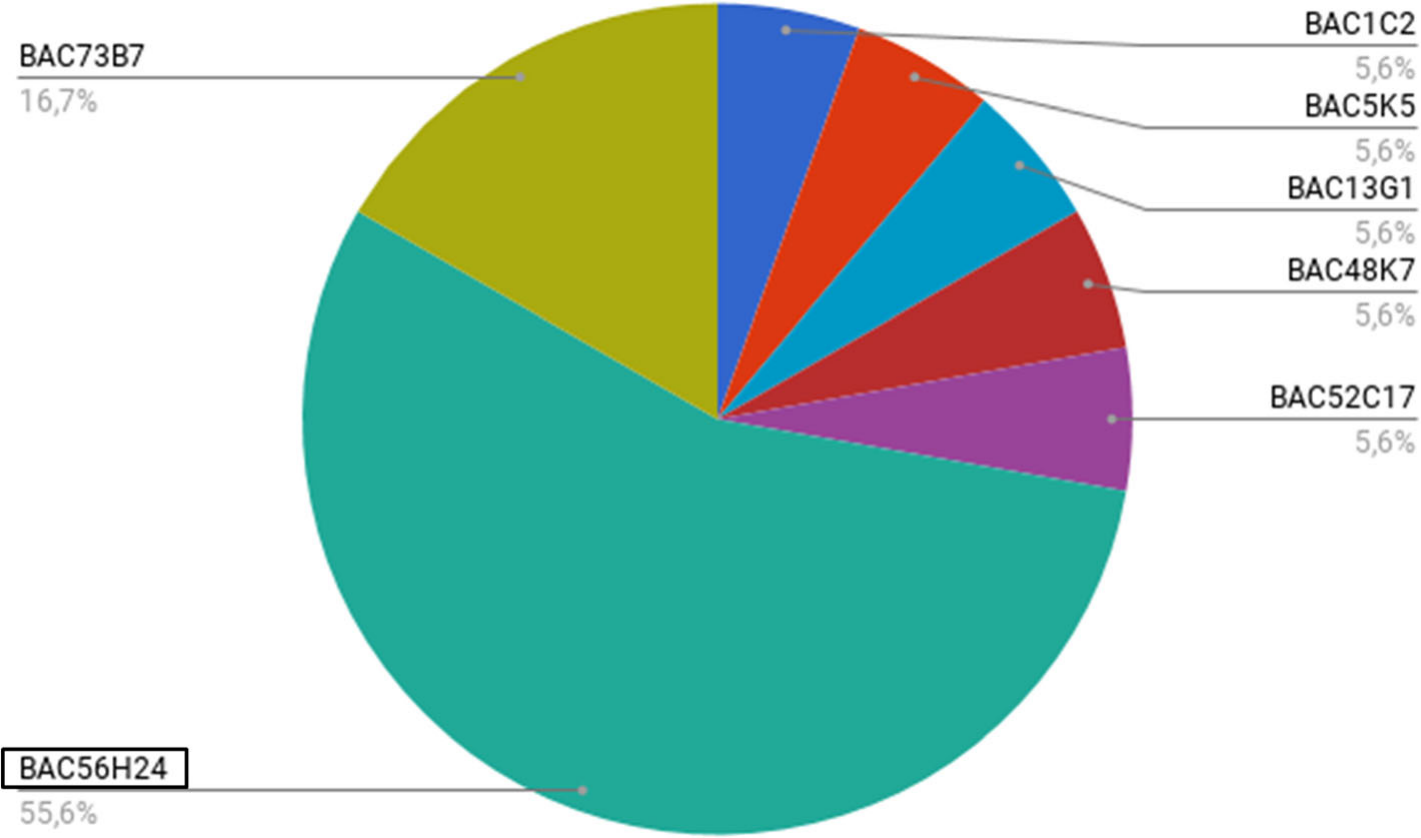
Relative content of satellite DNA in seven out of the 13 BAC clones used for this work. The remaining six BAC clones did not contain satellite sequences

The comparison of each BAC gene array localization between *S. senegalensis* and *C. semilaevis* demonstrated that 60% of the arm 1 genes were distributed mainly between chromosome 2 (20%) and chromosome 3 (40%) of *C. semilaevis* (Fig. 3b and Additional file 2). The remaining genes were distributed among chromosomes 6 (2.7%), 14 (5.7%), 20 (11.4%) and Z (14.3%); however, 5.7% of genes could not be found in any chromosome. Concerning arm 2, the genes were mainly distributed between chromosomes 19 (35.3%) and chromosome 20 (52.9%). The remaining two genes were either localized in chromosome 3 or not localized. The genes of BAC56H24 localized in the centromere of *S. senegalensis* were mainly localized in chromosome 18 of *C. semilaevis*, except for one localized in chromosome 5 and another that could not be found. After analyzing the repetitive elements within each BAC, this centromeric BAC clone showed the highest content in satellite DNA (Fig. 5), specifically different repetitions of the ONSATB satellite family. Other repetitive elements showed normal values (Additional file 3).

**Fig. 6:**
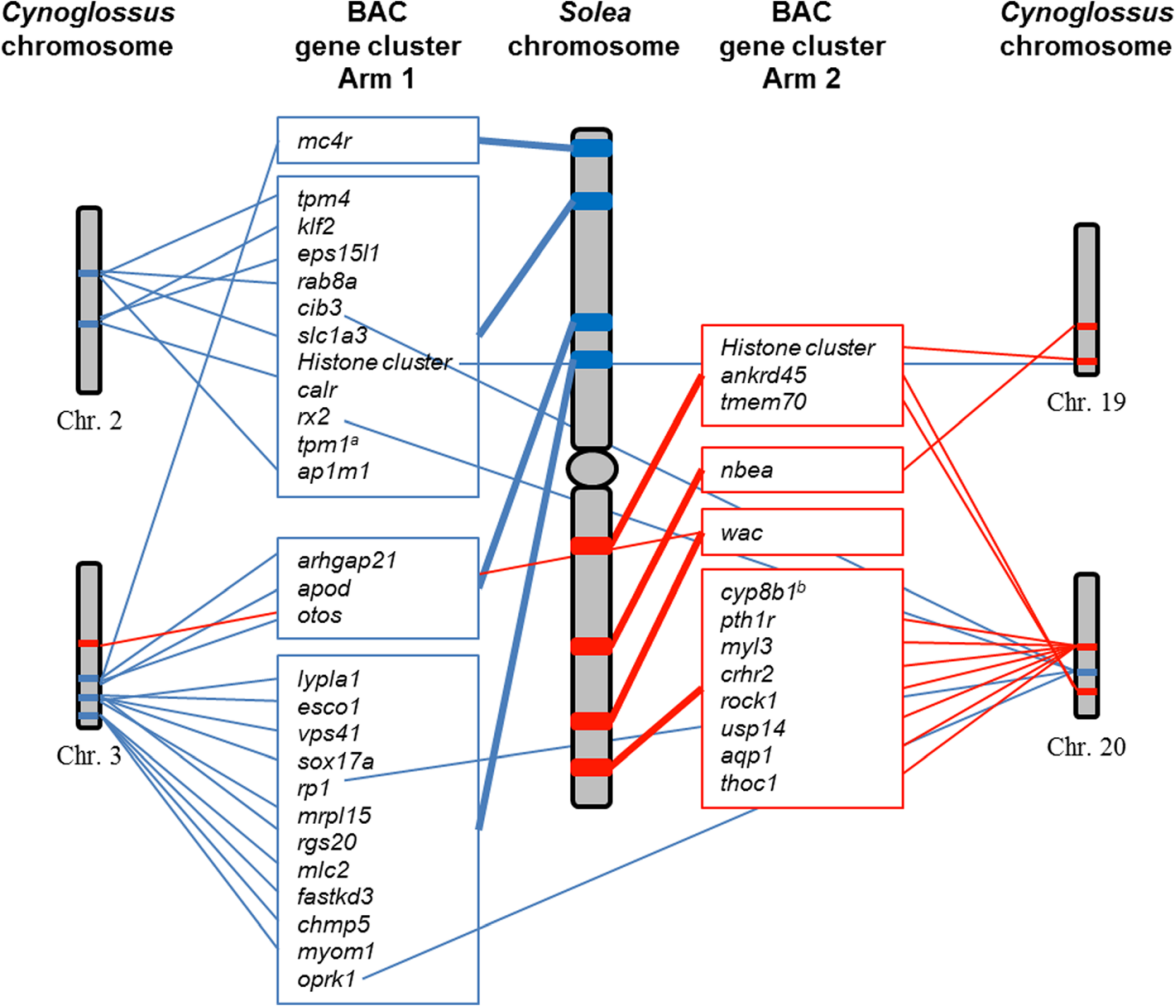
Localization of the genes contained within BAC clones in the chromosomes 2, 3, 19 and 20 of *Cynoglossus semilaevis*. Blue lines show the localization of the *Solea senegalensis* arm 1 genes in the chromosomes of *C. semilaevis*. Red lines show the localization of the *S. senegalensis* arm 2 genes in the chromosomes of *C. semilaevis*

Taking into account the chromosomes of *C. semilaevis* that share more genes with respect to the large metacentric pair of *S. senegalensis*, i.e., chromosomes 2 and 3 for arm 1 and chromosomes 19 and 20 for arm 2, several rearrangements between the two species can be observed (Fig. 6). A translocation and/or inversion event has been detected with the *tpm4*-*rab8a*-*slc1a3-ap1m1* and *klf2*-*eps15l1*-*calr* genes from the same BAC clone. The *mc4r* gene from BAC36D3 has also been localized at a position similar to that of BAC73B7, thus indicating a translocation event. In addition, some genes from BAC clones localized in arm1 (*cib3*, *rx2*, *rp1* and *oprk1*) have been detected in the same position of the chromosome 20 of *C. semilaevis*, which is an expected location for genes of arm 2 in the chromosome of *S. senegalensis.* The opposite has been observed with BAC13G1 localized in arm 2, in which case the unique gene (*wac*) was localized in chromosome 3 of *C. semilaevis*, which is an expected location for genes of arm 1 in the chromosome of *S. senegalensis* (Fig. 6).

## Discussion

As already stated, the Pleuronectiformes order is a taxonomically-complex group in which Robertsonian events could have played an important role as evolutionary mechanisms during the speciation of this group of fishes [9, 30, 31]. Recently, this kind of event has been proposed as having caused the appearance of the large metacentric chromosome in *S. senegalensis* [11]. However, there is no conclusive evidence for such a particular Robertsonian fusion and it is not clear if the event occurred during the evolution of the Soleidae family or if it arose earlier during the radiation of the Pleuronectiformes.

It could not be ascertained whether BAC56H24 belongs to arm 1 or arm 2, because it hybridized in the centromeric region, and this BAC clone showed a large content in satellite DNA. It has been reported that repetitive elements of this kind represent the major DNA component of many centromeric regions [32]. The satellite DNA found in BAC56H24 matches the ONSATB satellite family described in the fish *Oreochromis niloticus* [33] and it was localized on the centromeres of this fish species and also scattered throughout the chromosome arms [34]. Moreover, BAC56H24 also showed centromeric hybridization signals on subtelocentric and acrocentric pairs, so this satellite family is specific for three chromosome pairs of *S. senegalensis*. The organizational pattern of the repeat-based centromeres differs among the species [32] and the pattern of *S. senegalensis* probably comprises satellite DNA families specific to different chromosomes. Indeed, new satellite DNA was isolated recently in three species of the *Solea* genus and hybridized in the majority of *S. senegalensis* chromosomes, except in two pairs [35].

It is known that teleost fishes have undergone three rounds of whole genome duplications (WGD) [36] and, as a result, the duplicated genes could have suffered a sub- or a neo-functionalization event [37]. As observed in this study (Additional file 1) and in previous BAC-FISH analyses carried out in *S. senegalensis* [11, 19, 20], when a BAC clone shows more than one signal, normally one of them is stronger than the other. This does not occur with BAC56H24, since the three signals are of similar intensity; therefore, they are probably due to a specific duplication of the centromeric satellite DNA involving these three chromosome pairs rather than the WGD. In addition, the comparative analysis of the BAC56H24 genes with those of *C. semilaevis* indicated that such genes are localized within chromosome 18, which is not one of the main chromosomes that share genes with the large metacentric chromosome of *S. senegalensis*. Hence, both Zoo-FISH (the probe did not paint the centromeres) and comparative genome analysis (satellite DNA located in chromosome 18 in *C. semilaevis* but arm 1 and 2 of *S. senegalensis* are related to chromosomes 2, 3 and 19, 20, respectively) point to the same result with respect to the different origin of the centromeres compared with the origin of the chromosome arms.

In this study, the karyotype of two Soleidae species, *D. cuneata* and *D. lusitanica*, has been described for first time. This shows a chromosome complement comprising 2n = 50 and 2n = 42, respectively. To date, the karyotype is known in 11 species of the Soleidae family, and variability in diploid number can be observed (Table 2). Although the karyotype of 42 chromosomes seems to be the most shared diploid number (5 out of the 11 species), more species might to be studied to definitively conclude that 2n = 42 chromosomes is the plesiomorphic condition for the Soleidae family, above all when this number has been observed almost exclusively within the *Solea* genus. The 2n = 50 chromosome complement observed in *D. cuneate* has been described for first time in the Soleidae family [38–40].

**Table 2:** Diploid and fundamental chromosome numbers in species of the Soleidae family studied so far

Species	2n	FN	References
*Achirus lineatus*	40	64	[55]
*Dagetichthys lusitanica*	42	50	present study
*Dicologlossa cuneata*	50	54	present study
*Heteromycteris oculus*	48	54	[56]
*Microchirus ocellatus*	42	56	[57]
*Solea lascaris*	42	56–58	[15]
*Solea lascaris nasuta*	42	48	[41]
*Solea lutea*	30	44	[42]
*Solea senegalensis*	42	60	[35]
*Solea solea*	42	56–58	[15]
*Trinectes maculatus*	40	50	[55]
*Zebrias zebra*	46	46	[43]

A complement of 2n = 48 acrocentric chromosomes has been proposed as the ancestral karyotype for Pleuronectiformes, based on that being the karyotype observed in the most species of the sister group, the Carangidae family; it is also observed in the majority of flatfish species studied so far [9]. However, from this ancestral karyotype, a considerable reduction of chromosome number and increase of arm number have been observed across the different families of the Pleuronectiformes order (reviewed by [9]), which could be explained by Robertsonian fusions and pericentromeric inversions, for chromosomes and arms respectively. It has been postulated that the reduction in the diploid number within a group of species is related to life lived in specialized or constant habitats [41]; therefore the adaptation to a specialized and constant benthic lifestyle in Pleuronectiformes is consistent with this assumption. *D. cuneata* represents an exception, given the increase in karyotype by two chromosomes with respect to the predicted ancestral karyotype of Pleuronectiformes. Chromosome fission is a plausible way for this diploid number to have been reached in *D. cuneata*, as has already been proposed for fish species of the genus *Rhabdolichops* of the Gymnotiformes order [42].

It has been proposed that the large metacentric chromosome of *S. senegalensis* is derived from a Robertsonian fusion [20]. The large metacentric painting probe hybridized in four acrocentric chromosomes from two different species of the Soleidae family, namely *D. cuneata* and *D. lusitanica*, thus confirming the Robertsonian fusion theory. In addition, the centromeric regions of these acrocentric chromosomes were not painted, probably due to the existence of different repetitive families within the centromeres of these chromosomes. The existence of a Robertsonian fusion has also been demonstrated in another flatfish species, *Trinectes inscriptus*, by the existence of Internal Telomeric Sequences (ITS) in a metacentric pair [30]. No ITS regions were localized in *S. senegalensis* [17], so probably a progressive loss of the ITS could have occurred after a Robertsonian fusion. However, the absence of ITS could be due to the number of copies of the telomeric sequence being insufficient for the FISH technique to be capable of detecting [43] or to a telomere loss that occurred prior to the robertsonian fusion [44]. The differences in diploid number between *S. senegalensis* and *D. cuneata* could be explained by fusion and fission events respectively. However, *D. lusitanica* and *S. senegalensis* both have 2n = 42 chromosomes, so the diploid number of these two species must come from different fusion pathways. Furthermore, the two species differ in the number of arms (FN = 60 and FN = 50, for *S. senegalensis* and *D. lusitanica*, respectively), thus indicating the occurrence of more complex chromosomal rearrangements in *S. senegalensis*, such as inversions or translocations. This difference in the fusion pathways followed by the Pleuronectiformes species could be a useful tool to help to resolve the complex taxonomy of the group, as has already been proven for resolving phylogenetic relationships in rodents [45] and bovid species [46]. Diversification and sex chromosome origins by independent chromosome fusions have been studied in fish species of the *Eigenmannia* genus [47]. The *dmrt1* gene was localized in the large metacentric chromosome of *S. senegalensis* [20], in addition to the canonical histone cluster [11]. The location of multi-gene families in sex chromosomes has also been reported in some other species [48]. *Dmrt1* and its duplicates have proposed as sex-determining genes in many species [49], including the closely-related species *C. semilaevis* [50]. These findings in *S. senegalensis* have led to the large metacentric chromosome being proposed as a proto-sex chromosome [20]; therefore sex chromosome painting probes could also be applied to corroborate this proposal definitively and to ascertain the chromosome evolution within the Pleuronectiformes.

A reduction in diploid number is explained by chromosome fusions, but the reduction in arm number is not. This situation was clearly demonstrated in two closely-related species of the Mugilidae family, in which *Mugil rubrioculus* has 2n = 48 acrocentric chromosomes (FN = 48) whereas *Mugil curema* has 2n = 24 bi-armed chromosomes (FN = 48) [51]. The karyotype of *C. semilaevis* comprises 2n = 42 acrocentric chromosomes (FN = 42); therefore Robertsonian fusions followed by pericentric inversions could account for this karyotype.

The comparative genomic analysis between *S. senegalensis* and *C. semilaevis* has provided evidence that arm 1 of the large metacentric chromosome of *S. senegalensis* shares genes mainly with chromosomes 2 and 3 of *C. semilaevis*, whereas arm 2 shares genes mainly with chromosomes 19 and 20 (see Fig. 4 for more details). Therefore, the evolutionary transition among these four chromosomes of *C. semilaevis*, the two pairs observed in *D. cuneata* and *D. lusitanica*, and the large metacentric chromosome of *S. senegalensis*, could be due to tandem and Robertsonian fusion events, or to Robertsonian fusions followed by peri- and paracentric inversions. Indeed, chromosome rearrangement events of all these kinds are found to have determined the trends of evolution in both deer and cattle species [52].

Comparing the positions of the BAC clones within the large metacentric chromosome, with respect to the four chromosomes in *C. semilaevis*, it can be observed that genes that were localized together in the same locus of *C. semilaevis*, were separated in *S. senegalensis*; this observation is evidence of both translocations and pericentric and paracentric inversions within the large metacentric chromosome. In a previous study, this kind of rearrangement was also proposed to explain the localization of two clusters of canonical histones in different arms of the large metacentric chromosome, demonstrated at both molecular and cytogenetic level [11]. Therefore, substantial rearrangement activity must have occurred during the evolution of this particular metacentric chromosome.

It has been stated that sex chromosomes differ from autosomes by having undergone more complex chromosomal rearrangements [52]. As an example, the sex chromosomes of neotropical fishes were proven to have arisen by Robertsonian and tandem fusions [53]. Inversion events have also been stated to be an essential step for suppressing recombination between proto-sex chromosome homologues [54]. Therefore, both rearrangements (Robertsonian fusion and inversions) have been associated with the emergence of proto-sex chromosomes. These two rearrangements have also been observed in the large metacentric chromosome of *S. senegalensis*, thus reinforcing the proto-sex chromosome theory of this chromosome pair. However, further analyses are necessary to evaluate the accumulation of repetitive elements and the absence of recombination between chromosome homologues.

## Conclusions

In this study, a Zoo-FISH technique has been carried out in a flatfish species for first time. The results obtained from this, and from a comparative genomic analysis, have demonstrated that the large metacentric chromosome of *S. senegalensis* has originated from a Robertsonian fusion of two acrocentric chromosomes homologues of this metacentric chromosome. Events producing intensive reorganizations have been detected in this chromosome. As a consequence, new clues about the evolutionary pathway of the Pleuronectiformes order have been traced, and this work establishes this group of fishes as a model species for research into chromosomal rearrangement. Further analysis of cross-species hybridization, including more Pleuronectiformes species, needs to be undertaken in order to elucidate more exactly the karyotype and sex chromosome evolution in this taxonomically-complex group.

## Additional files

Additional file 1: Chromosome localization of the 13 BAC clones within the large metacentric chromosome of *Solea senegalensis*. (PPTX 703 kb)

Additional file 2: BAC clone localization within each arm of the *Solea senegalensis* large metacentric chromosome, localization and function of each annotated gene among chromosomes of *Cynoglossus semilaevis*. (DOCX 19 kb)

Additional file 3: Relative content in different repetitive elements within each BAC clone. (PPTX 193 kb)

## Abbreviations

AR: Arm Ratio
BAC: Bacterial Artificial Chromosome
CI: Centromeric Index
DEAC: Diethylaminocoumarin fluorochrome
DOP-PCR: Degenerate Oligonucleotide Primed – Polymerase Chain Reaction
EST: Expressed Sequence Tag
FISH: Fluorescence in situ Hybridation
FITC: Fluorescein isothiocyanate fluorochrome
FN: Fundamental Number
ITS: Internal Telomeric Sequences
LB: Luria Bertani broth
NCBI: National Center for Biotechnology Information
ONSATB: satellite family
RL: Relative Length
SCI – CM: Central Research Services in Marine Culture, University of Cádiz
SO: Spectrum Orange fluorochrome
TR: Texas Red fluorochrome
WGD: Whole Genome Duplications
Zoo-FISH: cross-species chromosome painting: Zoo Fluorescence in situ Hybridation
