# Supplementary material for: Evidence for a Robertsonian fusion in Solea senegalensis (Kaup, 1858) revealed by zoo-FISH and comparative genome analysis
Source: BMC Genomics. 2018 Nov 14;19:818. doi: 10.1186/s12864-018-5216-6 (PMC6236887; doi:10.1186/s12864-018-5216-6)
Supplement: Supplementary file 1 — Chromosome localization of the 13 BAC clones within the large metacentric chromosome of Solea senegalensis. (PPTX 703 kb) [file 12864_2018_5216_MOESM1_ESM.pptx]

## Slide 1
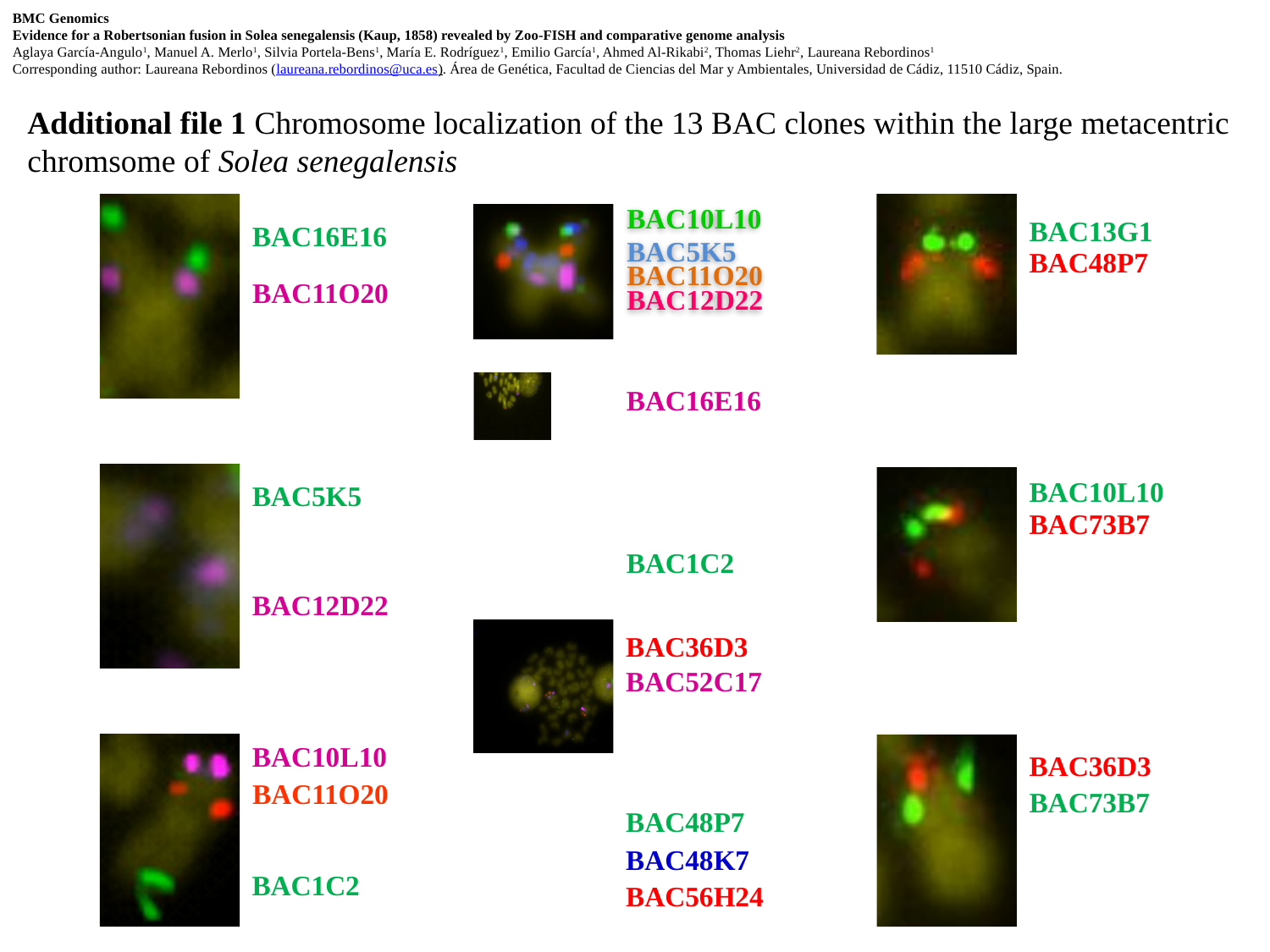

BMC Genomics
Evidence for a Robertsonian fusion in Solea senegalensis (Kaup, 1858) revealed by Zoo-FISH and comparative genome analysis
Aglaya García-Angulo1, Manuel A. Merlo1, Silvia Portela-Bens1, María E. Rodríguez1, Emilio García1, Ahmed Al-Rikabi2, Thomas Liehr2, Laureana Rebordinos1
Corresponding author: Laureana Rebordinos (laureana.rebordinos@uca.es). Área de Genética, Facultad de Ciencias del Mar y Ambientales, Universidad de Cádiz, 11510 Cádiz, Spain.
Additional file 1 Chromosome localization of the 13 BAC clones within the large metacentric chromsome of Solea senegalensis
BAC16E16
BAC11O20
BAC10L10
BAC5K5
BAC11O20
BAC12D22
BAC13G1
BAC48P7
BAC16E16
BAC1C2
BAC5K5
BAC12D22
BAC10L10
BAC73B7
BAC36D3
BAC52C17
BAC10L10
BAC11O20
BAC1C2
BAC36D3
BAC73B7
BAC48P7
BAC48K7
BAC56H24
