# Supplementary material for: Evidence for a Robertsonian fusion in Solea senegalensis (Kaup, 1858) revealed by zoo-FISH and comparative genome analysis
Source: BMC Genomics. 2018 Nov 14;19:818. doi: 10.1186/s12864-018-5216-6 (PMC6236887; doi:10.1186/s12864-018-5216-6)
Supplement: Supplementary file 2 — BAC clone localization within each arm of the Solea senegalensis large metacentric chromosome, localization and function of each annotated gene among chromosomes of Cynoglossus semilaevis. (DOCX 19 kb) [file 12864_2018_5216_MOESM2_ESM.docx]

**BMC Genomics**

**Evidence for a Robertsonian fusion in *Solea senegalensis* (Kaup, 1858) revealed by Zoo-FISH and comparative genome analysis**

Aglaya García-Angulo^1^, Manuel A. Merlo^1^, Silvia Portela-Bens^1^, María E. Rodríguez^1^, Emilio García^1^, Ahmed Al-Rikabi^2^, Thomas Liehr^2^, Laureana Rebordinos^1^

Corresponding author: Laureana Rebordinos (laureana.rebordinos@uca.es). Área de Genética, Facultad de Ciencias del Mar y Ambientales, Universidad de Cádiz, 11510 Cádiz, Spain.

**Additional file 2** BAC clone localization within each arm of the *Solea senegalensis* large metacentric chromosome and localization and function of each annotated gene among chromosomes of *Cynoglossus semilaevis*.

| **BAC** | **Gene annotation** | **Chromosome arm** | **Chromosome localization in**  ***Cynoglossus semilaevis*** | **Function** |
| --- | --- | --- | --- | --- |
|  |  |  |  |  |
| BAC1C2 | *nbea* | 2 | 19 | cell structure |
| BAC5K5 | *tpm4*  *klf2*  *eps15l1*  *rab8a*  *cib3*  *slc1a3*  *H2a*  *H3*  *H4*  *H2b*  *H1*  *calr*  *rx2*  *tpm1*  *ap1m1* | 1 | 2  2  2  2  20  2  19  19  19  19  19  2  20  6  2 | muscle contraction  transcription regulator  endocytosis  exocytosis  protein stability  nervous signaling  DNA structure  DNA structure  DNA structure  DNA structure  DNA structure  immune system  eye development, vision  muscle contraction  immune system |
| BAC10L10 | *klf2*  *eps15l1*  *rx2*  *calr* | 1 | 2  2  20  2 | transcription regulator  endocytosis  eye development, vision  immune system |
| BAC11O20 | *arrdc3*  *aqp3*  *nol6* | 1 | 14  14  ? | homeostasis  osmoregulation  rRNA processing |
| BAC12D22 | *H2a*  *H3*  *H4*  *H2b*  *H1*  *ankrd45*  *tmem70* | 2 | 19  19  19  19  19  20  20 | DNA structure  DNA structure  DNA structure  DNA structure  DNA structure  ?  mt ATP synthase biogenesis |
| BAC13G1 | *wac* | 2 | 3 | DNA damage response |
| BAC16E16 | *dmrt2*  *dmrt3* | 1 | Z  Z | cell differentiation and development  cell differentiation and development |
| BAC36D3 | *mc4r* | 1 | 3 | homeostasis and somatic growth |
| BAC48K7 | *dmrt3*  *kank1*  *dmrt1*  *fbp1*  *cfap157*  *dmrt2* | 1 | Z  Z  Z  Z  ?  Z | cell differentiation and development  regulator different biologic. process  sex determination  gluconeogenesis  spermatogenesis  cell differentiation and development |
| BAC48P7 | *cyp8b1*  *pth1r*  *myl3*  *crhr2*  *rock1*  *usp14*  *aqp1*  *thoc1* | 2 | ?  20  20  20  20  20  20  20 | bile acid synthesis  skeletal development  muscle contraction  stress response  cell structure  different catabolic processes  osmoregulation  transcription regulator |
| BAC52C17 | *lypla1*  *esco1*  *vps41*  *sox17a*  *rp1*  *mrpl15*  *rgs20*  *mlc2*  *fastkd3*  *chmp5*  *myom1*  *oprk1* | 1 | 3  3  3  3  20  3  3  3  3  3  3  20 | fatty acid metabolism  cell division and protein acetylation  endocytosis and exocytosis  embryogenesis  eye development  translation  regulator transduction signal  cardiac muscle contraction  cellular respiration  endocytosis and exocytosis  muscle contraction  behaviour |
| BAC56H24 | *akap9*  *hcn4*  *aqp10*  *hax1*  *tuft1*  *ubap2l*  *znf687b*  *C1orf43*  *pip5k1a* | centromeric | 18  5  18  18  18  18  ?  18  18 | regulator different biologic. process  muscle contraction  osmoregulation  apoptosis regulator  bone mineraliz. and odontogenesis  hematopoietic stem cell homeosta.  transcription  oxidoreductase activity  cell structure and migration |
| BAC73B7 | *arhgap21*  *apod*  *otos* | 1 | 3  3  3 | Golgi organization  lipid metabolic process  sensory perception of sound |
