# Supplementary material for: Evidence for a Robertsonian fusion in Solea senegalensis (Kaup, 1858) revealed by zoo-FISH and comparative genome analysis
Source: BMC Genomics. 2018 Nov 14;19:818. doi: 10.1186/s12864-018-5216-6 (PMC6236887; doi:10.1186/s12864-018-5216-6)
Supplement: Supplementary file 3 — Relative content in different repetitive elements within each BAC clone. (PPTX 193 kb) [file 12864_2018_5216_MOESM3_ESM.pptx]

## Slide 1
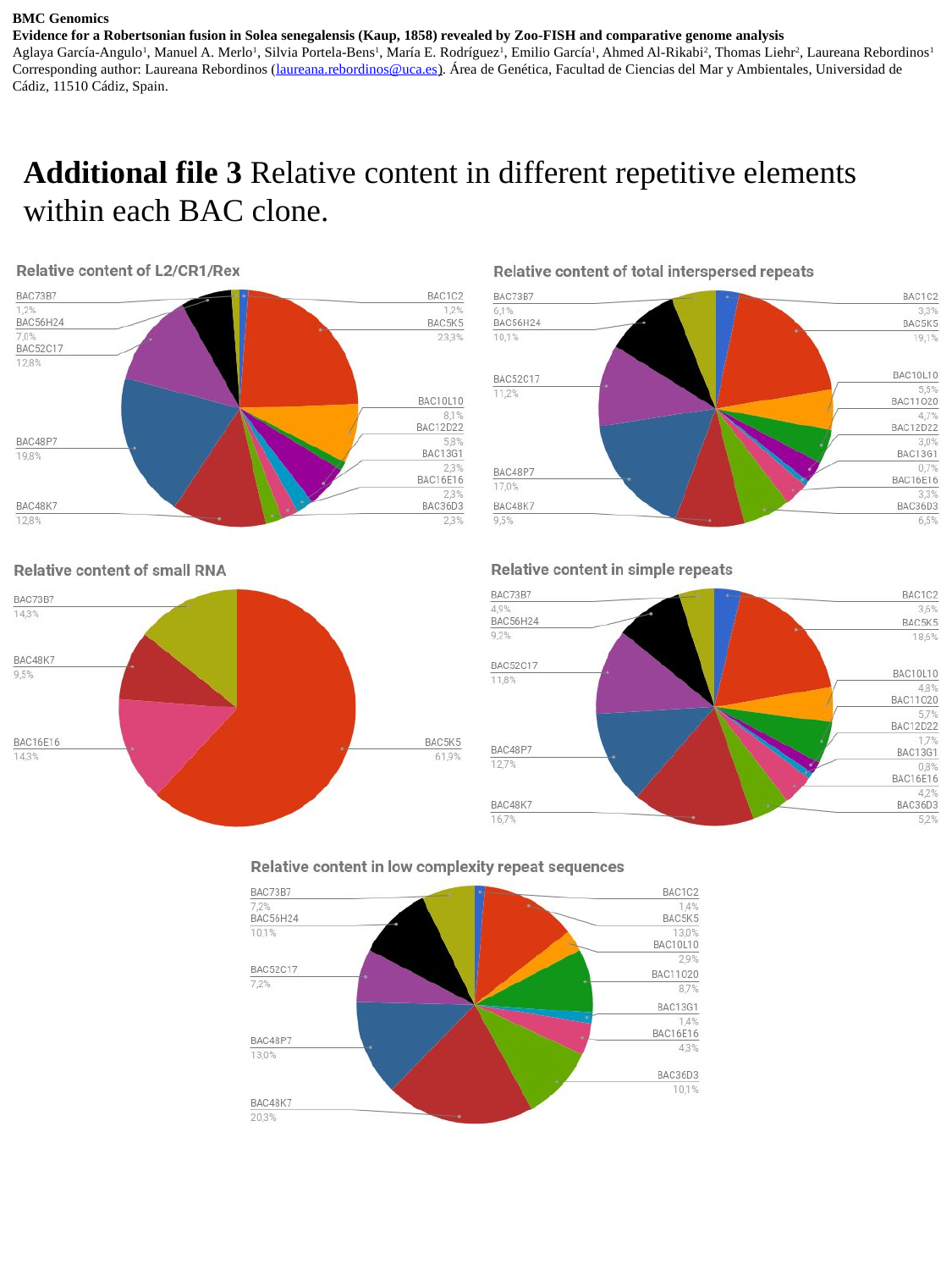

BMC Genomics
Evidence for a Robertsonian fusion in Solea senegalensis (Kaup, 1858) revealed by Zoo-FISH and comparative genome analysis
Aglaya García-Angulo1, Manuel A. Merlo1, Silvia Portela-Bens1, María E. Rodríguez1, Emilio García1, Ahmed Al-Rikabi2, Thomas Liehr2, Laureana Rebordinos1
Corresponding author: Laureana Rebordinos (laureana.rebordinos@uca.es). Área de Genética, Facultad de Ciencias del Mar y Ambientales, Universidad de Cádiz, 11510 Cádiz, Spain.
Additional file 3 Relative content in different repetitive elements within each BAC clone.
